# Expression of the schizophrenia associated gene *FEZ1* in the early developing fetal human forebrain

**DOI:** 10.3389/fnins.2023.1249973

**Published:** 2023-09-08

**Authors:** Maznah Alhesain, Hannah Ronan, Fiona E. N. LeBeau, Gavin J. Clowry

**Affiliations:** Centre for Transformative Research in Neuroscience, Newcastle University Biosciences Institute, Newcastle upon Tyne, United Kingdom

**Keywords:** axonogenesis, cerebral cortex, ganglionic eminences, neurodevelopmental diseases (NDDs), SNARE complex, thalamus, transcription

## Abstract

**Introduction:**

The protein fasciculation and elongation zeta-1 (FEZ1) is involved in axon outgrowth but potentially interacts with various proteins with roles ranging from intracellular transport to transcription regulation. Gene association and other studies have identified *FEZ1* as being directly, or indirectly, implicated in schizophrenia susceptibility. To explore potential roles in normal early human forebrain neurodevelopment, we mapped *FEZ1* expression by region and cell type.

**Methods:**

All tissues were provided with maternal consent and ethical approval by the Human Developmental Biology Resource. RNAseq data were obtained from previously published sources. Thin paraffin sections from 8 to 21 post-conceptional weeks (PCW) samples were used for RNAScope *in situ* hybridization and immunohistochemistry against *FEZ1* mRNA and protein, and other marker proteins.

**Results:**

Tissue RNAseq revealed that *FEZ1* is highly expressed in the human cerebral cortex between 7.5–17 PCW and single cell RNAseq at 17–18 PCW confirmed its expression in all neuroectoderm derived cells. The highest levels were found in more mature glutamatergic neurons, the lowest in GABAergic neurons and dividing progenitors. In the thalamus, single cell RNAseq similarly confirmed expression in multiple cell types. In cerebral cortex sections at 8–10 PCW, strong expression of mRNA and protein appeared confined to post-mitotic neurons, with low expression seen in progenitor zones. Protein expression was observed in some axon tracts by 16–19 PCW. However, in sub-cortical regions, *FEZ1* was highly expressed in progenitor zones at early developmental stages, showing lower expression in post-mitotic cells.

**Discussion:**

FEZ1 has different expression patterns and potentially diverse functions in discrete forebrain regions during prenatal human development.

## Introduction

The protein fasciculation and elongation zeta-1 (FEZ1) has been shown to be involved in axon and dendrite outgrowth (Chua et al., 2021), but potentially interacts with various proteins whose roles range from intracellular transport systems to transcription regulation (Razar et al., 2022). Gene association studies have directly identified *FEZ1* as a schizophrenia susceptibility gene (Yamada et al., 2004; Tang et al., 2017), although others have failed to replicate this finding (Hodgkinson et al., 2007; Koga et al., 2007; Nicodemus et al., 2010). FEZ1 knockout mice display hyperlocomotion and increased sensitivity to psychostimulants (Sakae et al., 2008) although there was no significant effect upon brain morphology or performance in a battery of cognitive tests. Nevertheless, its expression has been shown to be reduced in post-mortem brains of schizophrenia sufferers with mutations in *DISC1* (Disrupted in Schizophrenia 1; Lipska et al., 2006) as well as in peripheral blood of schizophrenia sufferers (Vachev et al., 2015). In addition, HDAC11 is a schizophrenia susceptibility gene (Kebir et al., 2014) knock down of which has been demonstrated to downregulate expression of *FEZ1* (Bryant et al., 2017). Kang et al. (2011) failed to find a significant direct association of variation within the *FEZ1* gene and risk for schizophrenia but instead found an epistatic interaction such that there is an approximate 2.5-fold increased risk for schizophrenia is seen in individuals carrying a *FEZ1* mutation but only in the context of a mutant *DISC1* background. Importantly, FEZ1 deficient stem cell derived human motoneurons show substantial developmental defects in axon growth and synapse formation *in vitro* (Gunaseelan et al., 2021) suggesting that human neurons may respond differently to rodent neurons in response to FEZ1 deficits.

FEZ1’s most well-characterized role is as a Kinesin-1 adaptor protein. Kinesin-1 is a motor protein that binds microtubules and directs the transport of protein assemblies or organelles along cell processes (Hirokawa and Noda, 2008). Adaptor proteins, such as FEZ1, regulate these transport processes by modulating and directing transport of specific cargoes (Hirokawa et al., 2009). FEZ1 has been demonstrated to be involved in the transport of multiple SNARE complex associated and axon growth associated proteins, e.g., syntaxin-1, SNAP25, synatoptagmin and GAP43, including others identified as neurodevelopmental disease susceptibility candidates, e.g., neurexins, STXBP1 and DISC1 (Toda et al., 2008; Kang et al., 2011; Chua et al., 2012; Butkevich et al., 2016; Razar et al., 2022). FEZ1 makes separate interactions with the Netrin-1 receptor, Deleted in Colorectal Cancer, and Semaphorin-3A receptor complex in the growth cone and thus may act downstream of guidance cues to regulate axon and dendrite outgrowth by directing delivery of cargoes to bring about expansion of processes by exocytosis involving SNARE proteins (Cotrufo et al., 2011; Chua et al., 2021). Clearly, alterations in function to FEZ1 could potentially disrupt axon and dendrite growth and formation and maintenance of synaptic networks.

However, FEZ1 is also expressed within the nucleus and has been demonstrated to interact with various transcriptional regulators (Teixeira et al., 2019). For instance FEZ1 interaction with short coiled protein (SCOC) as well as being implicated in axon growth and autophagy (Toda et al., 2008; McKnight et al., 2012; Qinlin et al., 2022) has also been shown to play a role in induction of expression of the neural transcription factor SOX2 (Papanayotou et al., 2008). It has also been demonstrated that FEZ1 interacts with the retinoic acid receptor and in the presence of retinoic acid brings about a dramatic increase in expression of the transcription factor HOXB4 (Teixeira et al., 2018). FEZ1 may have an important role to play in regulating the transcriptional program of neurons and their progenitors.

Therefore, it can be hypothesized that mutations in *FEZ1* or its interacting partners may contribute to the manifestation of neurodevelopmental diseases by disturbing the early trajectory of forebrain development through disruption of axon/dendrite outgrowth and early synapse formation, as well as possibly altering gene expression programs controlling cell phenotype specification. In order to test these hypotheses, it is necessary to first establish expression of *FEZ1* in development, in which regions and cell types and at what developmental time points. In the present study, we have combined data mined from gene expression databases with histological investigations in human tissue samples to provide a description of *FEZ1* mRNA and protein expression in the developing forebrain.

## Materials and methods

### Human tissue

Human fetal tissue from terminated pregnancies was obtained from the joint MRC/Wellcome Trust-funded Human Developmental Biology Resource (HDBR; Gerrelli et al., 2015).^1^ All tissue was collected with appropriate maternal consent and approval from the Newcastle and North Tyneside NHS Health Authority Joint Ethics Committee. Fetal samples from genetically normal cases ranging in age from 7.5 to 21 PCW were used. Ages were estimated from foot and heel to knee length measurements according to Hern (1984).

### RNAScope *in situ* hybridization

RNA *in situ* hybridization experiments were performed using the RNAscope^®^ technology, which has been previously described (Wang et al., 2012). Paired double-Z oligonucleotide probes were designed and manufactured by ACD Bio Techne against target RNA (HsFEZ1; catalogue number 468471, a 20ZZ probe targeting base pairs 255–1,433 of NM_005103.4). The *RNAscope* Reagent Kit (ACD Bio Techne, Abingdon, United Kingdom) was used according to the manufacturer’s instructions but with slight modifications. In brief, 8 μm-thick paraffin sections were baked on a heating pad for 10 minutes at 60°C, dewaxed in xylene, and then boiled with target retrieval buffer (ACD) for 20 min at 95°C. Protease digestion was carried out at 40°C for 30 min, followed by probe hybridization for 2 h at 40°C with target probes. The hybridized signals were amplified by a cascade of signal amplification molecules and detected with the RNA*scope* 2.5 HD detection kit (Fast Red). Slides were counterstained with toluidine blue, positive signals showed as red chromogenic dots in the cytoplasm or nucleus. Each sample was quality controlled for RNA integrity with a probe specific to the housekeeping gene *GAPDH*. Negative control background staining was evaluated using a probe specific to the bacterial *dapB* gene.

### RNAscope fluorescent *in situ* hybridization coupled with immunofluorescence

*In situ* hybridization was carried out first as described above except that the hybridized signals were amplified and detected using Opal 570 tyramide signal amplification (TSA; Akoya Biosciences, Delaware, United States). Then immunofluorescent staining was carried out according to previously described protocols (Alzu’bi et al., 2022) with various antibodies (Table 1). Sections were boiled in 10 mM citrate buffer pH 6 for 10 min, followed by incubation with primary antibody (diluted in 10% normal blocking serum in Tris buffered saline [TBS] pH 7.6) overnight at 4°C. Sections were then incubated with HRP-conjugated secondary antibody for 30 min (ImmPRESS^™^ HRP IgG [Peroxidase] Polymer Detection Kit, Vector Labs). Signals were detected with Opal 520 TSA applied for 10 min (Akoya Biosciences). Sections were mounted using Vectashield antifade mounting medium with DAPI (Vector Labs, Peterborough, United Kingdom). Sections from eight fetal brains ranging in age from 8 to 21 PCW were used.

**Table 1:** Primary antibodies employed.

Primary antibody to	Species	Dilution	Supplier	RRID number
FEZ1	Rabbit polyclonal	1:500	Proteintech, Manchester, United Kingdom	AB_2877825
Doublecortin (DCX)	Rabbit polyclonal	1:500	Abcam, Cambridge, United Kingdom	AB_732011
TBR1	Rabbit polyclonal	1:1,000	Abcam.	AB_2200219
Ki67	Mouse monoclonal	1:500	Santa Cruz, Heidelberg, Germany	AB_627859
GAD67	Mouse monoclonal	1:1,000	Merck Millipore, Watford, United Kingdom	AB_2278725
GAP43	Rabbit polyclonal	1:500	Novus Biologicals, Bio Techne, Abingdon, United Kingdom	AB_10001196

## Results

### *FEZ1* transcriptomics in the human forebrain

**Figure 1:**
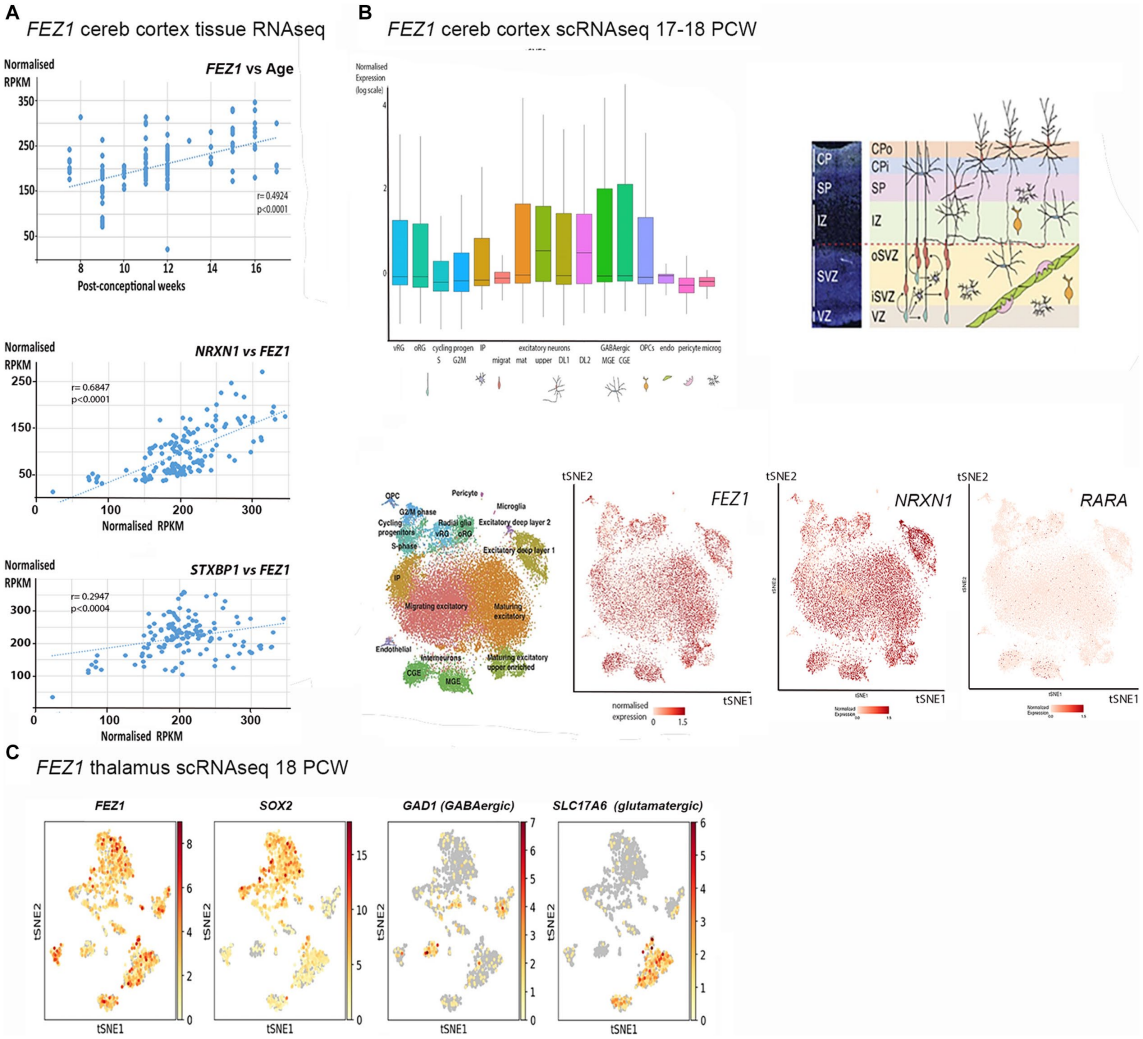
*FEZ1* expression: transcriptomic analysis. **(A)** Whole tissue RNAseq performed on 138 cortical samples at various developmental stages (post-conceptional weeks PCW). Expression levels given as normalized RPKM. Expression of *FEZ1* increases significantly with age and is highly correlated with expression of *NRXN1* and *STXBP1*. **(B)** Single cell RNAseq data for cerebral cortex at 17/18 PCW taken from solo.bmap.ucla.edu/shiny/webapp/. **(A)** t-distributed stochastic neighbor embedding (t-SNE) map and accompanying bar charts revealed that *FEZ1* is ubiquitously expressed in neuroectoderm derived cell types, but especially in more mature excitatory (glutamatergic) neurons, less mature GABAergic neurons (CGE and MGE) and non-dividing radial glia. Expression was lower in dividing progenitor cells and more mature GABAergic neurons (interneurons). *NRXN1* exhibited a very similar distribution of expression, whereas *Retinoic acid receptor A (RARA)* exhibited lower and more restricted expression. **(C)** single cell RNA data for thalamus at 18 PCW taken from NeMO Analytics. T-SNE maps generated for *FEZ1* and a marker for progenitor cells (*SOX2*), GABAergic neurons (*GAD1*, GAD67) and glutamatergic neurons (*SLC17A6*, vGLUT2) revealed that *FEZ1* was also ubiquitously expressed in thalamus at this stage of development.

A number of databases were consulted to provide information on *FEZ1* expression, including whole tissue and single cell RNAseq. Using data deposited at www.ebi.ac.uk/arrayexpress/experiments/E-MTAB-4840 (Lindsay et al., 2016). 138 samples of cortical tissue taken at ages ranging from 7.5 to 17 PCW, and from various positions along the anterior posterior axis of the cortex including the temporal lobe were analyzed for tissue RNAseq. As shown in Figure 1A, *FEZ1* was highly expressed from the beginning of cortical plate formation (7.5 PCW) until the latest developmental stage studied, with expression increasing significantly with age. For all samples except one expression levels are in the top quartile for protein coding genes (> 40 normalized RPKM) and in 85% of samples expression levels were in the top 5% of protein coding genes (> 160 normalized RPKM; Harkin et al., 2017). This suggests a crucial role for FEZ1 in cortical development, and that it might be predominantly expressed in post-mitotic cells, which increase as a proportion of the cell population in the cortical wall with age. No statistically significant difference in expression between cortical regions was observed (not shown). This suggests that FEZ1 does not play a role in cortical arealisation.

To explore the cell type specificity of *FEZ1* expression further, the cortical development expression viewer (CoDEx)^2^ a scRNAseq database, was interrogated. This provides data on expression from human cortical tissue samples at 17/18 PCW (Polioudakis et al., 2019). As shown in Figure 1B, *FEZ1* was found to be ubiquitously expressed in a high proportion of cells, including glutamatergic and GABAergic neurons, as well as subtypes of progenitor cell. Expression was consistently highest in more mature glutamatergic neurons (subplate and lower cortical plate) lower in dividing progenitor cells compared to quiescent progenitors, and lowest in cells of non-neuroectodermal origin such as microglia, pericytes and endothelial cells. Expression levels were highly variable in many cell types.

We also examined expression of genes for proteins known to interact with FEZ1 (see Introduction). For tissue RNAseq, we found two neurodevelopmental susceptibility genes that also interact with the SNARE complex and showed the same pattern of high and increasing expression with time (Figure 1A). We found that *neurexin 1 (NXRN1)* expression, a high probability susceptibility gene for schizophrenia (Hu et al., 2019) was particularly tightly coupled to *FEZ1* expression across our samples. *STXBP1* expression was also highly correlated with *FEZ1*, but not as tightly as *NRXN1*. *STXBP1* has been associated with schizophrenia (Behan et al., 2009) although with less evidence than *NRXN1* but is more commonly associated with infantile epilepsy (Xian et al., 2022). On the other hand, the synapse associated susceptibility gene *DISC1* showed only low levels of expression which did not change over time (not shown). We further examined scRNA seq for the cortex. This confirmed findings of high expression for *NRXN1* (Figure 1B) and *STXBP1* (not shown) with a strikingly similar cellular distribution to *FEZ1*. *In situ* hybridization data deposited at the BrainSpan atlas of the developing human brain (Ding et al., 2022)^3^ confirmed this expression pattern for *NRXN1* at 15 and 21 PCW with particularly high expression in mature glutamatergic neurons.

Retinoic acid receptors interact with FEZ1 at the level of transcriptional control (see introduction) we found there expression to be relatively low and uniform across development by tissue RNAseq (not shown). Cellular distribution of the most highly expressed isoform of retinoic acid receptor A (*RARA*) showed highest expression in quiescent radial glia and GABAergic interneurons or their immature precursors (Figure 1B) suggesting that FEZ1 may act to regulate transcription in these cell types.

Altered development of thalamocortical circuits are strongly implicated in schizophrenia (Jiang et al., 2021) thus cell specific expression in the thalamus was explored using data from an 18 PCW human thalamus (sample deposited at NeMO Analytics; Orvis et al, 2021). TSNe plots were constructed for *FEZ1* and markers of various cell phenotypes (Figure 1C) revealing that FEZ1 is also widely expressed in the thalamus, co-localizing with both glutamatergic (*SLC17A6*/vGLUT2 expressing) and GABAergic (*GAD1*/GAD67 expressing) neurons as well as progenitor cells and astrocytes (*SOX2* expressing).

Taken together, it appears that *FEZ1* is expressed by maturing neurons in certain locations, where it may play a role in axon, dendrite and synapse formation (see introduction) but is also widely expressed in progenitor cells, where it may regulate transcriptional programs but possibly play a role in extension of radial glial fibers.

### RNAScope *in situ* hybridization and immunohistochemical studies of *FEZ1* expression

**Figure 2:**
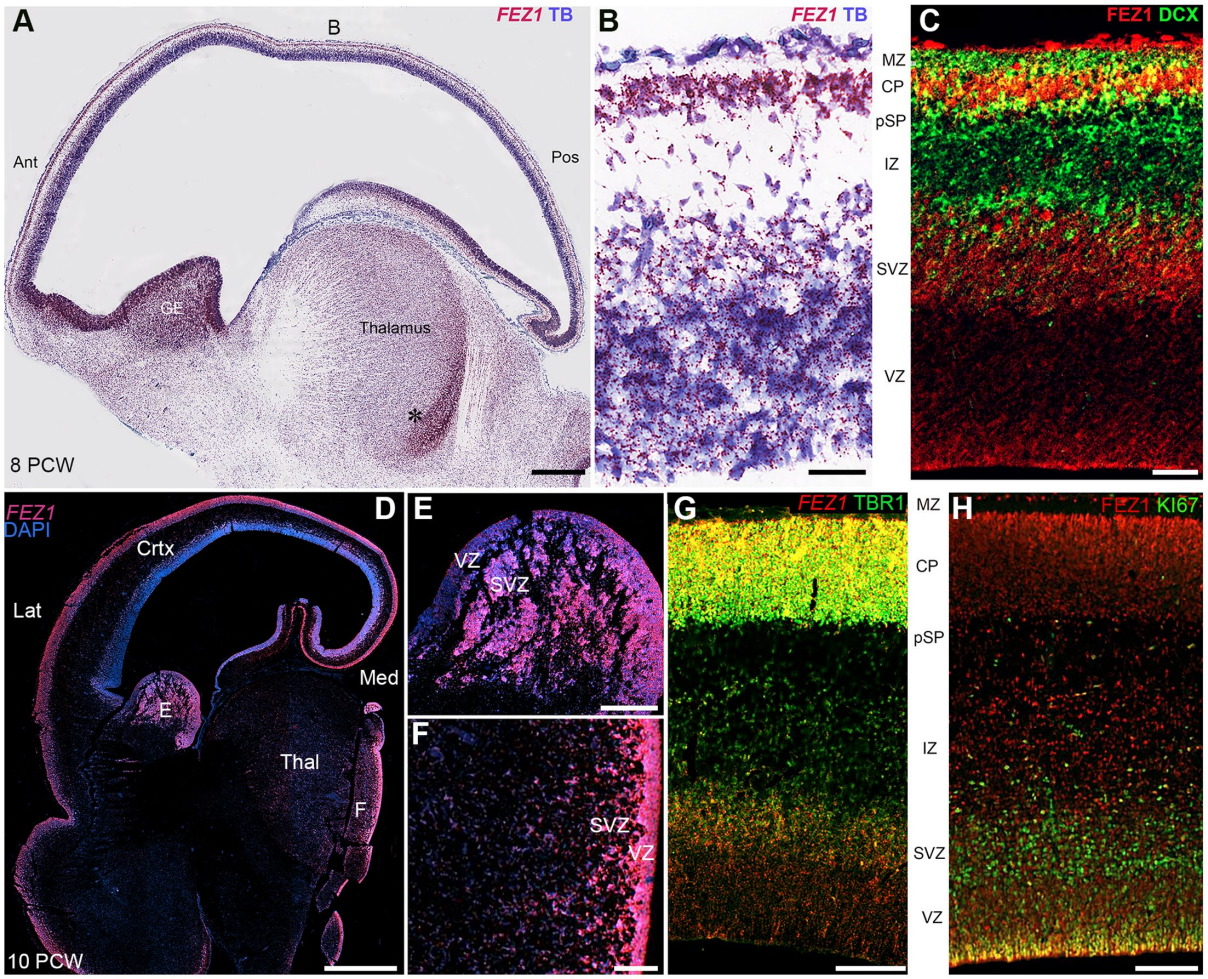
*FEZ1* expression: RNAscope and immunofluorescence 8–10 postconceptional weeks. **(A)** Sagittal section (8PCW) shows *FEZ1* mRNA expression throughout the forebrain, but strongest in the ganglionic eminences and proliferative zones of the thalamus (counterstained with toluidine blue TB). **(B)** shows the dorsal neocortex at higher magnification. FEZ1 expression was highest on the cortical plate (CP) and there was moderate expression in the ventricular and subventricular zones (VZ and SVZ). **(C)** double immunofluorescence staining for FEZ1 protein and the immature neuron marker doublecortin (DCX). FEZ1 was strongly expressed in the CP and also in the VZ and SVZ. There was co-expression (yellow) with DCX in the CP but not in the marginal zone (MZ) presubplate (pSP) or intermediate zone (IZ). **(D)** coronal section (10 PCW) confirms continued expression of FEZ1 at this developmental stage and the alternate pattern of stronger post-mitotic neuron expression in the cortex, and stronger progenitor zone expression in the ganglionic eminences and thalamus (*). **(E)** (ganglionic eminences) and **(F)** (thalamus) mark the location of higher magnification panels. **(G)** fluorescent RNAScope/immunofluorescence staining for *FEZ1* and the post-mitotic neuron marker TBR1 and shows strongest co-expression (yellow) in the cortical plate. **(H)** double immunofluorescence staining for FEZ1 and cell division marker KI67. Strong co-expression (yellow) seen in cells on the apical (ventricular) surface, but hardly any co-expression in the SVZ. Ant, anterior; Lat, lateral; Med, medial; Pos, posterior; Thal, thalamus; Scale bars A, D, 1 mm; E, F, 200 μm; G, H, 100 μm; C, 50 μm.

In the cerebral cortex at 8 post conceptional weeks (PCW) just after the onset of cortical plate formation, expression of both *FEZ1* mRNA and FEZ1 protein was highest in the post-mitotic neurons of the subplate (SP) and cortical plate (CP), but expression was detectable in the progenitor cell containing ventricular and subventricular zones VZ and SVZ (Figures 2A–C). Protein expression was higher in neurons (identified by doublecortin immunoreactivity) settled in the cortical plate compared to those migrating through the subplate and intermediate zone suggesting these is no FEZ1 expression in DCX+ migrating neurons and neurites found in these locations (Figure 2C). However, in the proliferative ganglionic eminences (GE) and progenitor zones of the diencephalon (asterisk; Figure 2A) expression is higher than in sub-cortical post-mitotic neurons. This dichotomy is maintained at 10 post-conceptional weeks (Figures 2D–F). Interestingly *FEZ1* mRNA expression in dorsal and lateral cortical progenitor zones appeared decreased compared to medial zones, although this was not supported by immunohistochemical detection of FEZ1 protein expression (not shown). *FEZ1* mRNA was strongly co-localized with TBR1, a marker for post-mitotic glutamatergic neurons, but also expressed in non-TBR1 positive cells in the VZ and SVZ, presumably progenitor cells (Figure 2G). The transcriptomics studies (Figure 1) suggested actively dividing cortical cells show low levels of FEZ1 expression. Immunohistological evidence suggested that while putative dividing KI67+ progenitors in the SVZ show little evidence of FEZ1 co-expression, ventricular zone radial glial cells with cell bodies located at the apical surface (where they undergo mitosis; Taverna and Huttner, 2010) strongly co-express these proteins (Figure 2H).

**Figure 3:**
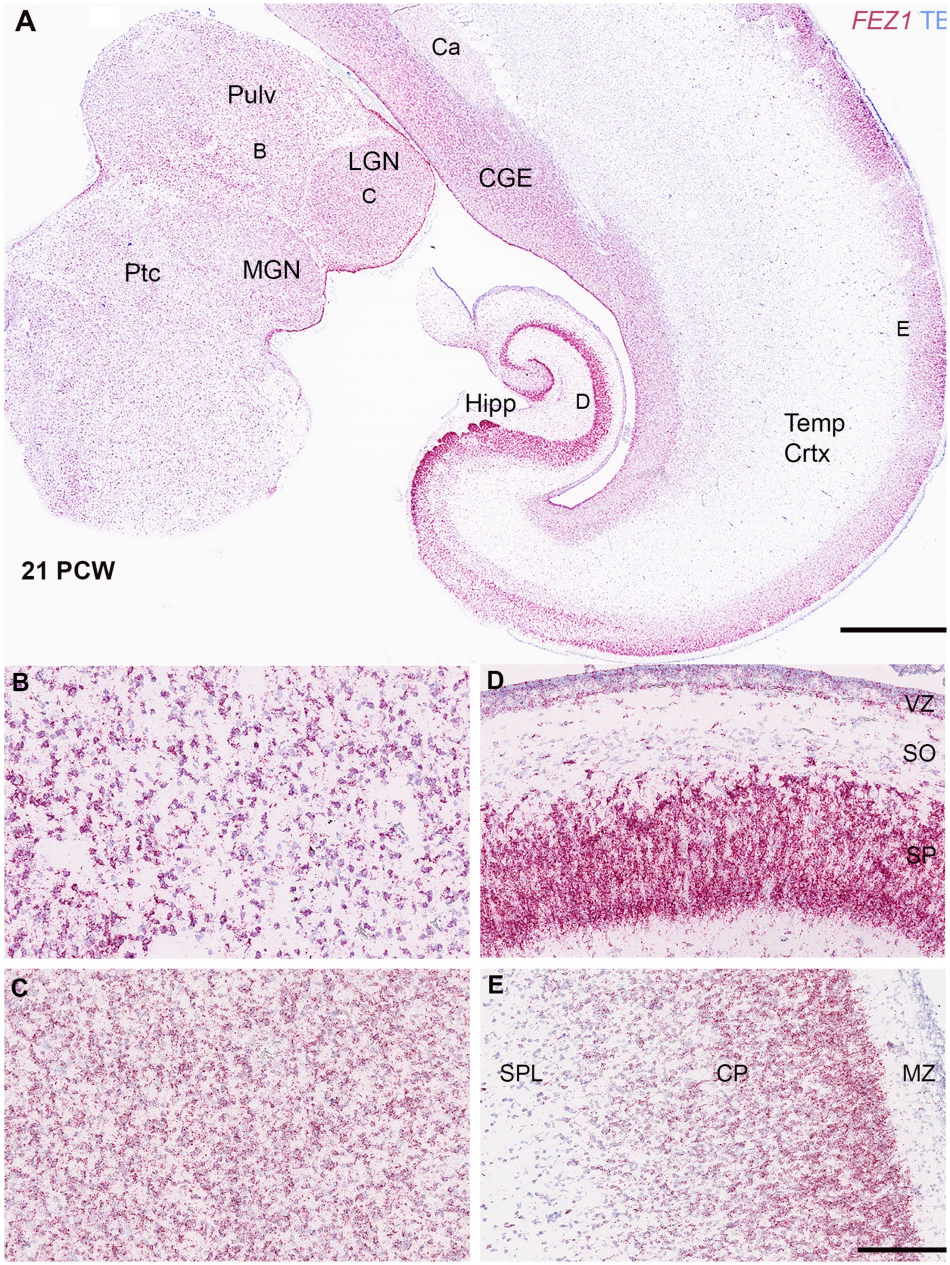
*FEZ1* expression: RNAscope at 21 postconceptional weeks. **(A)** coronal section showing temporal cortex (Temp Crtx), caudal ganglionic eminence (CGE) and thalamus (Pulv, MGN and LGN). At low magnification, expression was present in both progenitor and post-mitotic cell zones and positively correlated with cell density. However, expression appeared particularly dense in the hippocampus (Hipp) in the pyramidal cell layer. Higher magnification images of thalamus showed that the pulvinar region (Pulv; **B**) has a lower cell density but similar density of staining per cell compared to the Lateral Geniculate Nucleus (LGN; **C**). However, in cortical areas the hippocampal stratum pyramidalis (SP) which contains predominantly glutamatergic neurons there was very high expression per cell compared the stratum oriens (SO) which contains predominantly GABAergic neurons **(D)**. In the neocortex **(E)** expression was highest in the outer layers of the cortical plate (CP). Ca, caudate nucleus; MGN, medial geniculate nucleus; MZ, marginal zone; Ptc, pretectum; SPL, subplate. VZ, ventricular zone. Scale bars; A, 1 mm; B–E, 200 μm.

*FEZ1* mRNA expression was maintained in the forebrain throughout development and increased in the post-mitotic glutamatergic neurons of the thalamus. Different levels of expression were observed in different thalamic nuclei, with the lateral geniculate nucleus (LGN) showing higher levels of expression than the pulvinar, for instance (Figures 3A–C). However, this at least partially reflects a higher cell density in the LGN. Nevertheless, distinct thalamic nuclei are identifiable at this age unlike earlier stages of development shown in Figure 2.

Expression appeared strong in the cortical plate right up until the oldest stage studied (21 PCW; Figures 3A,E) and particularly in the pyramidal layer of the hippocampus (Figure 3D). Expression in the cortical plate was strongest at the boundary between the cortical plate and the marginal zone. This may partially reflect a higher density of cells at this location but also reflects higher levels of expression per individual cell. These neurons are the most recent cells to have stopped migrating to the cortical plate at this stage and thus this may suggest a role for FEZ1 in cell migration. This expression pattern has previously been observed for neurexins (Harkin et al., 2017; Ding et al., 2022) the intracellular transport of which is a mooted role for FEZ1 (see introduction). Intense expression of *FEZ1* by glutamatergic pyramidal neurons was a feature of the hippocampus, however, there was low expression in the stratum oriens which contains predominantly GABAergic neurons of various phenotypes (Figure 3A; Freund and Buzsáki, 1996). Similarly, the caudal ganglionic eminence, containing GABAergic progenitor cells, showed relatively high expression compared to the overlying caudate nucleus which is principally composed of post-mitotic GABAergic neurons. Relatively high levels of expression of *FEZ1* were still observed in the cortical VZ and SVZ although by this stage neurogenesis is ending and gliogenesis beginning (Cadwell et al., 2019) The VZ shows reduced levels of cell division at this stage (Nowakowski et al., 2016).

**Figure 4:**
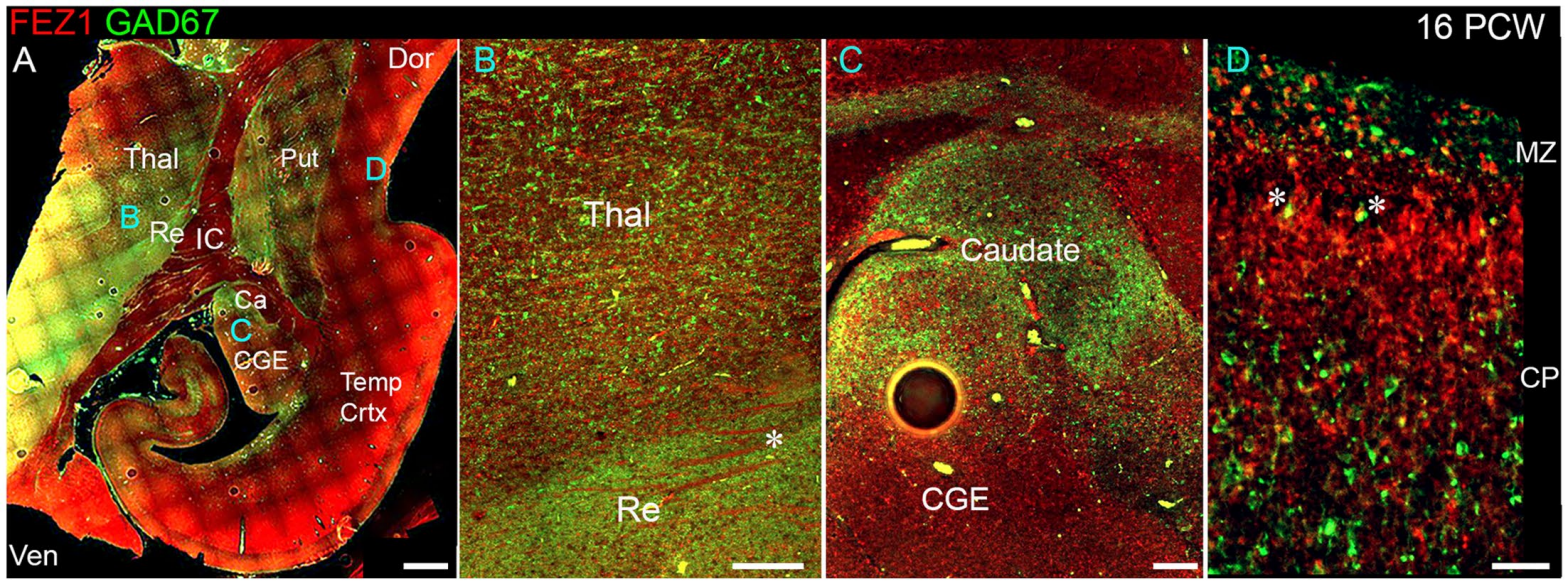
FEZ1/GAD67 immunofluorescence at 16 PCW. **(A)** Coronal section showing temporal cortex (Temp Crtx) basal ganglia and thalamus (Thal). FEZ1 protein expression was seen throughout the section, GAD67 expression was strongest in the thalamic reticular nucleus (Re) and subthalamic regions. FEZ1 was also moderately expressed in putamen (Put) caudate (Ca) ventral regions of the thalamus and cortical ventricular zone extending away from the CGE towards the hippocampal formation. **(B)** close up of the boundary between the thalamic reticular nucleus and the latero-ventral part of the thalamus. In both locations there was little evidence of co-expression of both markers in the same cell, although a higher proportion of red FEZ1+ cells can be seen in the thalamus. Also evident were bundles of FEZ1 positive axons (*) which are putative thalamocortical fibers (see Figure 5). **(C)** a higher magnification image of the caudal ganglionic eminence (CGE; progenitor zone for basal ganglia and GABAergic cortical neurons) and the caudate nucleus (Ca; containing predominantly GABAergic neurons) and illustrates how GABAergic neuron progenitors strongly expressed FEZ1 (red) but post-mitotic GAD67+ neurons (green) did not express FEZ1. Likewise, in the cortex **(D)** in the cortical plate (CP) GAD67+/FEZ1+ neurons were rare although isolated examples of double labelled cells are seen close to the border with the marginal zone (MZ, asterisks). Dor, dorsal; IC, internal capsule; Ven, ventral. Scale bars A, 1 mm; B, C, 200 μm; D, 100 μm.

We explored the apparent reduced expression of FEZ1 by GABAergic neurons further by examining co-expression of GAD67 (GABA synthesizing enzyme) and FEZ1 by immunohistochemistry at 16 PCW (Figure 4). The reticular nucleus of the thalamus, which in maturity comprises a sheet of GABAergic neurons surrounding the dorsal and lateral portions of the thalamus, showed high expression of GAD67, as expected, but showed little expression of FEZ1 (Figure 4B). Interestingly, there was a gradient of expression of GAD67 from ventral and lateral to dorsal parts of the thalamus (Figures 4A,B). Many FEZ1+ cell bodies were intermingled with GAD67+ cells in more ventral parts of the thalamus, but there was little evidence of co-expression. This suggests that thalamic post-mitotic thalamic glutamatergic neurons express FEZ1 by this stage of development. It also reveals potential pathways of migration of GABAergic neurons into the thalamus from the prethalamus (precursor of the reticular nucleus) and subthalamic midbrain regions at this stage of development. In other areas of high GABAergic cell density such as the nascent basal ganglia, GAD67 immunoreactivity was high but FEZ1 expression relatively low, although FEZ1 expression remained high in adjacent subpallial GABAergic neuroprogenitor zones (Figure 4C).

By 16 PCW GABAergic neurons have increased in the cortex having largely migrated there from the ganglionic eminences (Ma et al., 2013; Alzu’bi and Clowry, 2019). The majority of cortical GAD67+ neurons did not show double labelling for FEZ1 at 16 PCW, although in or near the marginal zone, a major pathway for migrating GABAergic neurons at this stage of development (Zecevic and Rakic, 2001; Meyer and González-Gómez, 2018) some small double-labelled cells were observed predominantly near or close to the MZ (Figure 4D). These may be the small calretinin positive GABAergic interneurons previously described (Meyer and González-Gómez, 2018; Alzu’bi and Clowry, 2020).

**Figure 5:**
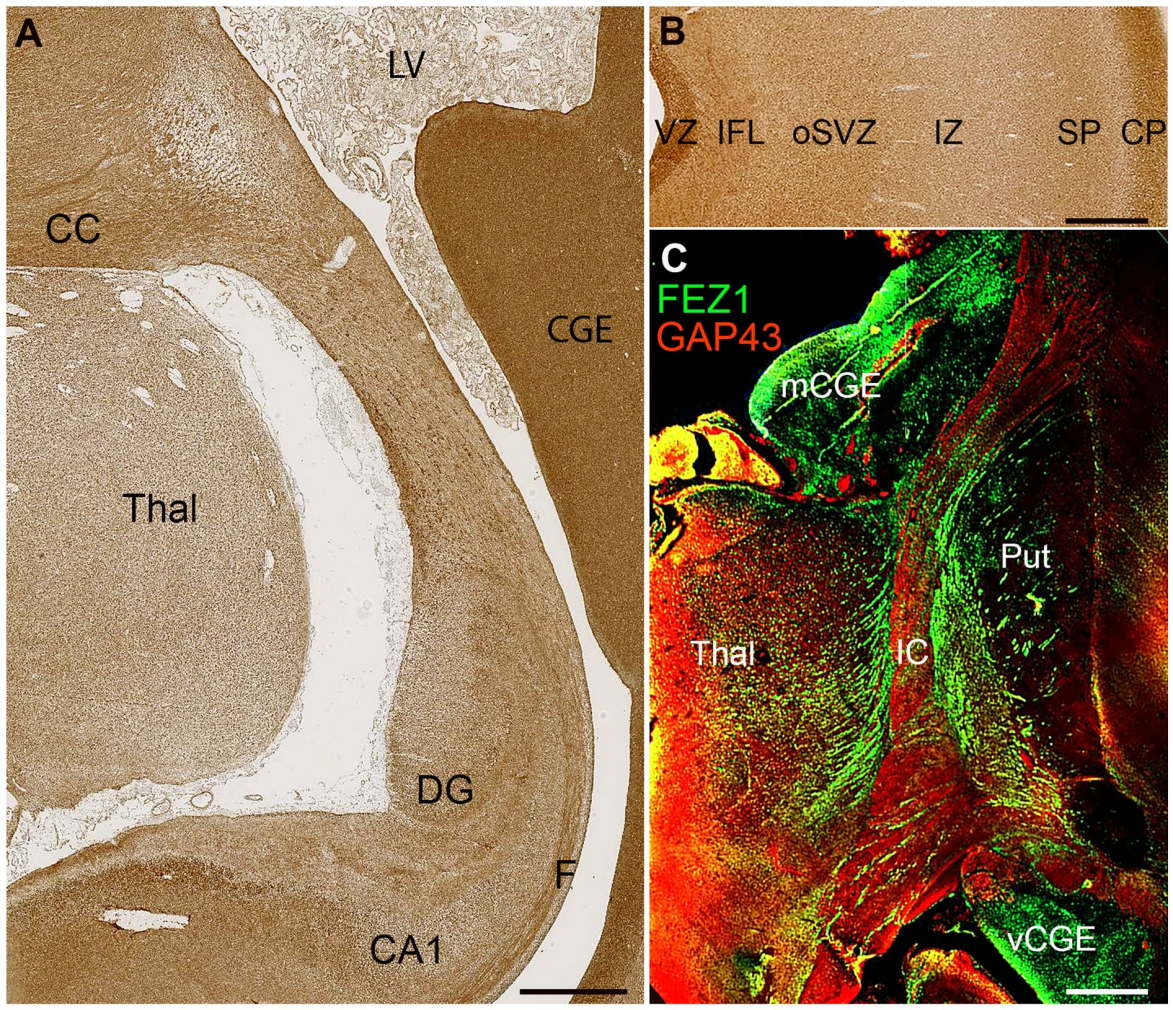
FEZ1 expression in axons. **(A)** posterior coronal section immunoperoxidase stained for FEZ1 at 19 PCW containing medial cortex, thalamus (Thal) and caudal ganglionic eminence (CGE). The hippocampal cortex is evident and axons in the fornix (F) are strongly immunoreactive. Axons in the corpus callosum (CC) are also strongly positive, as is the CGE, which contains GABAergic neuroprogenitor cells. **(B)** FEZ1 immunoreactivity in the lateral cortical wall; strong in the outer cortical plate (CP) and ventricular zone (VZ) moderate in the inner fiber layer (IFL) outer subventricular zone (oSVZ) and subplate (SP) and weak in the axon rich intermediate zone (IZ). **(C)** double immunofluorescence for FEZ1 and GAP43; coronal section, 16 PCW. GAP43 principally marks growing axons (Benowitz and Routtenberg, 1997) and in the internal capsule (IC) FEZ1 and GAP43 marked different bundles of axons suggesting FEZ1 may be expressed in fasciculating thalamocortical axons whereas GAP43 is expressed in growing corticofugal axons. FEZ1 was also strongly expressed in the proliferative medial and ventral caudal ganglionic eminences (mCGE and vCGE). Other abbreviations; CA1, cornu ammonis field 1; DG, dentate gyrus; LV, lateral ventricle; Put, putamen. Scale bars A, 1 mm; B, C, 500 μm.

FEZ1 protein expression at later developmental stages was not confined to neuronal cell bodies but showed selective enrichment in certain axon pathways. For instance, strong expression was observed in the fornix and certain callosal axons at 19 PCW (Figure 5A) but in the lateral cortical cortex, expression in the cortical intermediate zone, where large numbers of growing axons are found, was weak.

Relatively low FEZ1 expression in the subplate contrasted with stronger expression of both GAP43 and FEZ1 in cells of the cortical plate, and immunoreactivity in the inner fiber layer was less than in the ventricular zone (Figure 5B). Double labelling for the growing axon marker GAP43 and FEZ1 demonstrated a concentration of FEZ1 in selected axon pathways. For, instance, in the internal capsule at 16 PCW, FEZ1 was expressed by what appeared to be thalamocortical axons, whereas adjacent corticofugal axons were GAP43+ only (Figure 5C). We interpret this as showing that FEZ1 is selectively expressed by thalamocortical axons but more strongly in fasciculated axons proximal to the thalamus (Figure 5C) than in the distal terminal branches in the subplate (Figure 5B). FEZ1 expression is stronger in the cell bodies of glutamatergic neurons of the cortical plate than in their growing corticofugal axons in the internal capsule.

## Discussion

Analysis of transcriptomics data suggested that *FEZ1* is highly expressed in the developing human forebrain by a variety of cell types including progenitor cells and post-mitotic neurons. Histological studies confirmed this and revealed that while *FEZ1* was strongly expressed by cortical glutamatergic neurons and weakly expressed by their progenitors, the opposite is the case in the ventral telencephalon and diencephalon at early stages of development, where expression is much stronger in progenitor cells than in post-mitotic cells. FEZ1 is chiefly proposed to have roles in axon outgrowth, but its expression appeared to be stronger in some axon pathways than others. It also appeared that FEZ1 was strongly expressed in cell bodies of some cell groups and in the axons of other neurons. This suggests that FEZ1 has defined roles in development that are cell type specific. Our transcriptomic analysis strongly suggested that FEZ1 expression is closely coupled to expression of SNARE complex proteins also implicated in neurodevelopmental disorders. FEZ1 may play a significant role in transporting proteins such as NRXN1 and STXBP1 to sites of exocytosis in the developing cortex. SNARE mediated exocytosis not only plays a role in early synapse function, disruption of which could alter the forebrain’s developmental trajectory (Molnár et al., 2020) but also in adding plasma membrane to growing neurites at growth cones (Urbina and Gupton, 2020) again a vital process in early development.

Schizophrenia is considered a neurodevelopmental disorder in which many characteristic symptoms such as psychosis are exhibited in adolescence but other symptoms, such as deficits in working memory, are present from childhood. Therefore, it is conceivable that failures of cortical circuit formation at even earlier stages of development, including *in utero*, produce a susceptibility to later diseases manifestations as the brain matures. Disrupted thalamo-cortical-thalamic interactions have also been strongly implicated in schizophrenia (Woodward and Heckers, 2016; Anticevic and Halassa, 2023). Altered FEZ1 expression in thalamic progenitors may similarly affect differentiation of thalamic neurons. We also provide evidence for FEZ1 expression in post-mitotic thalamic neurons and their axons at later stages of development, suggesting that thalamic axon outgrowth and synapse formation may be affected.

On the other hand, in the cortex, FEZ1 expression seems more restricted to post-mitotic glutamatergic neurons and to the cellular rather axonal compartment (with the exception of hippocampal fornix projections). FEZ1 may have a role in dendrite and dendritic spine formation (see introduction) in this cell class. Hypofunctionality of pyramidal (glutamatergic) neurons in the cortex due to decreased dendritic spine and synapse formation has been implicated in schizophrenia (Glausier and Lewis, 2013) at least in certain cortical regions, for instance in the dorsal prefrontal cortex.

One group of cells consistently implicated in schizophrenia is cortical GABAergic interneurons (Glausier and Lewis, 2017). For instance, the schizophrenia susceptibility gene *ErbB4* is exclusively expressed by GABAergic neurons in the cerebral cortex in mice (Bean et al., 2014). Altered FEZ1 expression/activity in the progenitor cells of the ganglionic eminences may subtly alter the subsequent gene expression and differentiation of these cell types. Transcriptomic data presented here (Figure 1B) shows some potential for interaction of FEZ1 with retinoic acid receptors in GABAergic neurons or their precursors. The gene expression profiles of progenitor cell located in the ganglionic eminences is not explored here but there is evidence that some ganglionic eminence derived cells may migrate to the cortex but still retain the capacity to undergo cell division, and that the human cerebral cortex itself may produce some GABAergic neuron specific progenitors (Radonjić et al., 2014; Alzu’bi et al., 2017; Delgado et al., 2022).

We conclude that FEZ1 is expressed in multiple classes of forebrain neuron and neural progenitor and may have a number of roles dependent on timing, and spatial and cellular localization of expression. This may need to be studied further in human cell derived disease models in order to understand fully the potential roles FEZ1 plays in human neurodevelopment.

## Data availability statement

The datasets presented in this study can be found in online repositories. The names of the repository/repositories and accession number(s) can be found at: https://www.ebi.ac.uk/metagenomics/, E-MTAB-4840.

## Ethics statement

The studies involving humans were approved by Newcastle and North Tyneside NHS Health Authority Joint Ethics Committee. The studies were conducted in accordance with the local legislation and institutional requirements. The participants provided their written informed consent to participate in this study.

## Author contributions

GC, MA, HR, and FL: study design. MA and HR: data collection. GC, MA, FL, and HR: data interpretation. GC and MA: manuscript preparation. GC and FL: manuscript editing and finalization. All authors contributed to the article and approved the submitted version.

## Funding

The human fetal material was provided by the Joint UK MRC/Wellcome Trust funded (grants #099175/Z/12/Z and MR/R006237/1) Human Developmental Biology Resource (www.hdbr.org). The tissue RNAseq study was funded by a grant from UK MRC (grant #MC/PC/13047). MA is funded by a studentship from the Al Qassim University, Saudi Arabia.

## Conflict of interest

The authors declare that the research was conducted in the absence of any commercial or financial relationships that could be construed as a potential conflict of interest.

## Publisher’s note

All claims expressed in this article are solely those of the authors and do not necessarily represent those of their affiliated organizations, or those of the publisher, the editors and the reviewers. Any product that may be evaluated in this article, or claim that may be made by its manufacturer, is not guaranteed or endorsed by the publisher.

## Footnotes

^1^http://www.hdbr.org

^2^solo.bmap.ucla.edu/shiny/webapp/

^3^https://www.brainspan.org/

## Abbreviations

DAPI: 4′,6-diamidino-2-phenylindole
ErbB4: Erb-B2 Receptor Tyrosine Kinase 4
GABA: gamma amino butyric acid
GAD1: GABA decarboxylase 1
GAP43: growth associated protein 43 kDa
HDAC11: histone deacetylase 11
HOXB4: Homeobox B4
HRP: horse radish peroxidase
RNA: ribonucleic acid
SLC17A6: solute carrier family 17 member 6
SNAP25: synaptosome associated protein 25 kDa
SNARE: Snap Receptor
SOX2: SRY box transcription factor 2
STXBP1: syntaxin binding protein 1
vGLUT2: vesicular glutamate transporter 2
